# Language and movement disorders: early risk identification

**DOI:** 10.1186/1824-7288-40-S1-A66

**Published:** 2014-08-11

**Authors:** Francesca Pardo, Elena Vanadia

**Affiliations:** 1 “Io Comunico” Rehabilitation Center, Partinico (PA), Italy

## 

Psychomotor development is a maturational process that in the early years of life enables the child to acquire postural, motor, cognitive, communicative and relational skills and abilities. It is a continuous progress, essentially dependent on the maturation of the central nervous system (CNS), with variable timing and conditions for each child, but where is possible to identify the "stages" that are achieved in a similar sequence [1-6]. Knowledge of this "normal" development is needed to identify the early indicators of risk and to enable a project of "Care" which has as its objective the support of the child and its potential.

What was said can only occur in a network that sees Care and Rehabilitation Centers, Hospital and Territory, Pediatricians, School and Parents in close collaboration.

The therapeutic alliance Pediatrician-Child neurologist and psychiatrist-Parents is a key element for the evaluation and monitoring of the neurobehavioral development of the child and for the opportunity to do prevention. Through early diagnosis passes the opportunity to identify action habilitation / rehabilitation plans involving effective child and family, improving the knowledge of the natural history of specific neurodevelopmental disorders; ultimate goal is to guide the institutions on health policy interventions that enable an effective and efficient operation of the network [2].

The pathologies of language and movement are a "range of possibilities" with different gradations in the expressiveness, mirror of developmental system and brain mapping thatunderlies it, as well as the genetic control, we often see in comorbidity in different "individual trajectories”. Therefore about Autism Spectrum Disorders, specific learning disorders or dyspraxia, is to identify a large chapter with transitional symptom patterns that change in attitude and somehow coexist with variable expressivity in relation to the multigenic neurodevelopmental and environmental control that modulates them [3,4]

Is appropriate a reflection on broad spectrum screening models that, from the identification of early indicators of risk in the first years of life, allowing us to change the traditional model of rehabilitation of the child in relation to neurobiological model and individual evolutionary characteristics whose language and movement are an expression [5].
